# Development of genomic resources for the prairie vole (*Microtus ochrogaster*): construction of a BAC library and vole-mouse comparative cytogenetic map

**DOI:** 10.1186/1471-2164-11-70

**Published:** 2010-01-28

**Authors:** Lisa A McGraw, Jamie K Davis, Josh J Lowman, Boudewijn FH ten Hallers, Maxim Koriabine, Larry J Young, Pieter J de Jong, M Katharine Rudd, James W Thomas

**Affiliations:** 1 Center for Behavioral Neuroscience, Emory University, Atlanta, GA, USA; 2 Department of Human Genetics, Emory University School of Medicine, Atlanta, GA, USA; 3 Children's Hospital Oakland Research Institute, Oakland, CA, USA; 4 Department of Psychiatry and Behavioral Sciences, Emory University School of Medicine, Atlanta, GA, USA

## Abstract

**Background:**

The prairie vole (*Microtus ochrogaster*) is a premier animal model for understanding the genetic and neurological basis of social behaviors. Unlike other biomedical models, prairie voles display a rich repertoire of social behaviors including the formation of long-term pair bonds and biparental care. However, due to a lack of genomic resources for this species, studies have been limited to a handful of candidate genes. To provide a substrate for future development of genomic resources for this unique model organism, we report the construction and characterization of a bacterial artificial chromosome (BAC) library from a single male prairie vole and a prairie vole-mouse (*Mus musculus*) comparative cytogenetic map.

**Results:**

We constructed a prairie vole BAC library (CHORI-232) consisting of 194,267 recombinant clones with an average insert size of 139 kb. Hybridization-based screening of the gridded library at 19 loci established that the library has an average depth of coverage of ~10×. To obtain a small-scale sampling of the prairie vole genome, we generated 3884 BAC end-sequences totaling ~2.8 Mb. One-third of these BAC-end sequences could be mapped to unique locations in the mouse genome, thereby anchoring 1003 prairie vole BAC clones to an orthologous position in the mouse genome. Fluorescence in situ hybridization (FISH) mapping of 62 prairie vole clones with BAC-end sequences mapping to orthologous positions in the mouse genome was used to develop a first-generation genome-wide prairie vole-mouse comparative cytogenetic map. While conserved synteny was observed between this pair of rodent genomes, rearrangements between the prairie vole and mouse genomes were detected, including a minimum of five inversions and 16 inter-chromosomal rearrangements.

**Conclusions:**

The construction of the prairie vole BAC library and the vole-mouse comparative cytogenetic map represent the first genome-wide modern genomic resources developed for this species. The BAC library will support future genomic, genetic and molecular characterization of this genome and species, and the isolation of clones of high interest to the vole research community will allow for immediate characterization of the regulatory and coding sequences of genes known to play important roles in social behaviors. In addition, these resources provide an excellent platform for future higher resolution cytogenetic mapping and full genome sequencing.

## Background

Arvicoline rodents (including lemmings, muskrats and voles) represent a muroid lineage that split from the lineage leading to *Mus *and *Rattus *~24 million years ago (MYA) [1]. Amongst voles, the *Microtus *genus includes approximately 60 extant species that are primarily distributed across North America and Eurasia [2]. Based on fossil evidence, all members of the *Microtus *genus are hypothesized to be derived from a common ancestor that lived just ~2 MYA [3,4] and it has been noted that the rate of speciation required to generate 60 species in such a short time-frame are likely to have been at least 20-fold higher than expected in a typical mammalian lineage [5]. Consistent with this rapid rate of speciation, vole genomes display signatures of elevated rates of evolution. For example, in a European vole, *M. rossiaemeridionalis*, the mitochondrial genome appears to be evolving at a higher rate than all other mammalian taxa examined [5], and integrations of mitochondrial DNA (NUMT transfers) into the European vole nuclear genome have occurred at a much higher rate than in either mice or rats [6,7]. In addition, the diploid karyotypes of *Microtus *voles are quite variable and range from 2n = 17-64 [8], suggesting that many chromosomal rearrangements have been fixed in a very short amount of time. Indeed, Maruyama and Imai determined that *Microtus *had the highest rate of karyotype alterations when compared to other rodents [8]. Finally, this genus is highly enriched for unusual genomic and genetic properties associated with the X chromosome (reviewed in [9]). Together, these unusual features of the *Microtus *genomes make them a promising model for the study of genome evolution.

In addition to possessing intriguing genomic features, a North American vole species, the prairie vole (*Microtus ochrogaster*), has become an important animal model for understanding the genetic and neurobiological mechanisms that give rise to variation in social behaviors [10,11]. Unlike more traditional mammalian model organisms (e.g. mouse (*Mus musculus*) and rat (*Rattus norvegicus*)), prairie voles are highly affiliative, socially monogamous, form enduring social bonds between mates (pair bonds) and display extensive biparental care of offspring [10]. Comparative studies between the socially monogamous prairie vole and other closely related, non-monogamous, uniparental vole species within the genus *Microtus *have led to the identification of key genes and neurocircuitry that differentiate the social repertoires of these species. For example, the neuropeptide vasopressin is known to be involved in the regulation of social behavior in a variety of mammals [12]. However, comparative studies of monogamous and non-monogamous vole species led to the discovery that the distribution and density of vasopressin 1a receptors (V1aR) within the reward and reinforcement circuitry of the brain, but not the peptide itself, underlies differences between these species in a male's propensity to form pair bonds or to engage in parental care [13]. Furthermore, a length polymorphism in a microsatellite upstream of the transcription start site of *avpr1a*, the gene encoding V1aR, is associated with the differential expression of V1aR in prairie voles [14]. These and other findings gleaned from prairie vole research have already yielded remarkable parallels between the genetic regulation of social cognition and behavior in voles and man [15], and future studies in the prairie vole promise to further advance our understanding of the molecular underpinnings of human social behavior.

Despite the tremendous value of the prairie vole as a candidate species to begin to understand the unique features of genome evolution within the genus *Microtus *and as a model system for studying the relationship between the genome and the brain in generating social behaviors, a lack of genomic resources has limited the potential of this extraordinary model system. Here we report the development of a pair of genomic resources for the prairie vole: a prairie vole bacterial artificial chromosome (BAC) library, and a first-generation prairie vole-mouse comparative cytogenetic map.

## Results

### BAC library construction and characterization

DNA extracted from a kidney of a single male prairie vole was used to generate a BAC library consisting of 194,267 recombinant clones. Estimates of the insert size from a sample of clones from the library indicated that average insert size was 139 kb and that the vast majority of clones contain inserts in the 100-200 kb size range. Further information as to the insert size distribution and other library properties can be found online at http://bacpac.chori.org/library.php?id=481. Assuming a genome size typical for placental mammals, ~2.8 Gb, the theoretical genome coverage of this library is ~9.6×. To experimentally verify the clone-depth of the library and its utility for targeted physical mapping, we screened the library with n = 54 probes from 19 discrete locations in the genome corresponding to genes that have been established to be associated with social behavior in voles, or other species (see Additional file 1). Based on probe-content and restriction-enzyme fingerprint BAC contigs at the targeted loci we observed an average clone depth of 10.2×, which is very close to the depth of coverage predicted for the library.

### Generation and characterization of prairie vole BAC-end sequences

To initiate a small-scale sequence survey of the prairie vole genome we attempted to generate BAC-end sequences (BESs) from all clones corresponding to library plates #1-4, as well as those clones identified in the above targeted mapping efforts. In total, 3884 successful BESs were generated with an average quality-trimmed read length of 728 bp. Similar to other rodent genomes [15,16], the GC content of the prairie vole genome estimated based on the ~2.8 Mb of BES data was 41.8%. The repetitive element content of the prairie vole BESs as determined by RepeatMasker http://www.repeatmasker.org was just 22.2%, which is much lower than the ~42% repeat content observed other rodents [15,16]. However, the low observed repeat content in prairie voles compared to other rodents most likely reflects an inability to identify vole-specific repeats rather than a true compositional difference between the genomes. Finally, we did not detect any evidence of NUMT elements within the BESs, suggesting that while integration and duplication of these elements in the *Microtus *genome are elevated compared to other species [6,7], they are not a common wide-spread feature of the prairie vole genome.

### Comparative mapping of prairie vole BAC-end sequences

To begin to develop a prairie vole-mouse comparative map, we compared the prairie vole BESs to the mouse genome. Overall, we were able to map 33% (n = 1269) of the prairie vole BESs to a unique location in the mouse genome. As a result, 787 vole clones were anchored to the mouse genome via a single BES and an additional 241 vole clones via both BESs (Additional file 2, 3 &4). Of the clones in which both BESs mapped to the mouse genome the majority (n = 181) were classified as concordant, and therefore could be definitively assigned to a single orthologous location in the mouse genome. Another 35 clones that were classified as discordant because the distance between the paired BESs was smaller (45-86 kb) or greater (201-329 kb) than expected based on the BAC insert sizes, presumably due to local insertions or deletions, could also be assigned to a single orthologous location in the mouse genome. The remaining vole clones with discordant mapping results were associated with two potential large (> 2 Mb) indels or complex rearrangements, three potential inversions, and 19 potential translocations. However, because each potential rearrangement between the prairie vole and mouse genomes detected by this method are only supported by one or two BES mate-pairs these results should be considered provisional evidence for the location of structural differences between the prairie vole and mouse genomes. Finally, after taking into account the relative size of the assembled mouse chromosomes and the fact that the library was made from a male, the observed chromosomal distribution of mapped prairie vole BESs generated from library plates 1-4 (i.e. a random sampling of the library) did not differ significantly from a random distribution expected by chance (Chi-square = 0.3, df = 20).

### Construction of a first generation prairie vole-mouse comparative cytogenetic map

**Figure 1:**
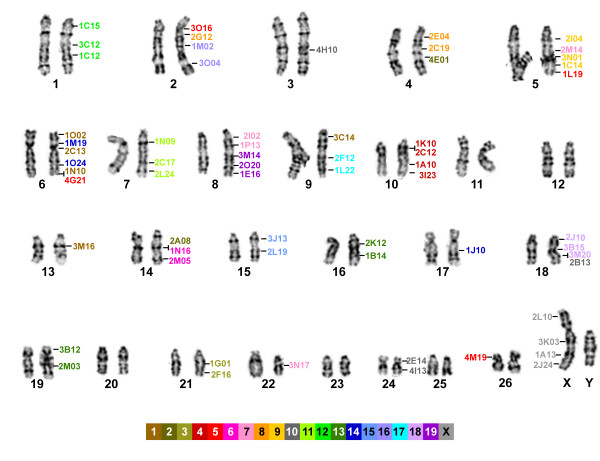
**A prairie vole-mouse comparative cytogenetic map**. The chromosomal positions of all successfully mapped prairie vole BAC clones are shown relative to the prairie vole karyotype. Names of the mapped clones are color-coded based on the mouse orthologous mouse chromosome, which is indicated by the color key at the bottom of the figure. Vertical lines indicate pairs of clones for which the relative position on the chromosome could not be resolved. Note that the chromosome numbering follows the prairie vole karyotype reported in [27].

**Figure 2:**
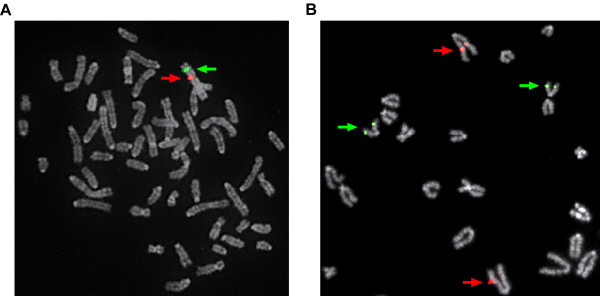
**Examples of FISH results in the prairie vole**. FISH was performed using pairs of prairie vole BAC clones orthologous to the same mouse chromosome hybridized to male vole metaphase spreads. Partial metaphase spreads are shown in both images. **A**) A pair of prairie vole BAC clones orthologous to the mouse X chromosome [CH232-3K03 (red signal) and CH232-1A13 (green signal)] are linked to the X chromosome in prairie vole. **B**) A pair of prairie vole clones orthologous to mouse chromosome 10 are unlinked in prairie vole and mapped to chromosome 3 (CH232-4H10, red signal) and chromosome 24 (CH232-4I13, green signal).

To generate a first generation prairie vole-mouse comparative map, we selected a set of 84 vole clones that were optimally spaced (~1 clone/30 Mb) across the genome (Additional file 5) and hybridized them to metaphase chromosome spreads from a male prairie vole. Though there was a significant hybridization failure rate amongst the clones (22/84 clones failed), prairie vole BACs orthologous to all mouse chromosomes with the exception of the Y, were represented in the comparative map, and at least one clone mapped to 21 of the 26 vole autosomes and the X chromosome (Figure 1). Of the 62 clones for which distinct hybridization signals were observed, most (n = 48) fell into groups of 2-4 probes that defined blocks of conserved synteny that ranged in size from ~23 Mb between vole and mouse chromosomes 15, to ~147 Mb between the vole and mouse X chromosomes (Figure 1, and for FISH example see Figure 2). The most extensive conserved synteny was observed between prairie vole chromosomes 1, 7, 10, 14, 15, 21, X and mouse chromosomes 12, 11, 4, 6, 15, 3, and X, respectively, in which all the mapped probes were linked to a single chromosome in both species. Among those and other instances of conserved synteny that were defined by 3-4 markers, the relative order of the markers was also conserved (conserved linkage) on prairie vole chromosomes 7, 8, 10 and 18 with mouse chromosomes 11, 19, 4 and 18, respectively. However, the relative order of markers differed between vole chromosomes and mouse chromosomes 12 and the X chromosomes, consistent with at least one inversion having occurred on both chromosomes since the most recent common ancestor of these rodents. In addition, the interdigitated order of markers orthologous to different mouse chromosomes on prairie vole chromosomes 5 and 6 were indicative of a minimum of one and two inversions, respectively being associated with those chromosomes (Figure 1 and Additional file 5).

Inter-chromosomal rearrangements that have occurred since the most-recent common ancestor of the prairie vole and mouse will have disrupted the physical linkage between markers resulting in orthology of a single prairie vole or mouse chromosome to two or more chromosomes in the other species. The most extreme examples of the evolutionary shuffling of the genome at the inter-chromosomal level were mouse chromosomes 1, 10, 7, each of which mapped to three vole chromosomes, and mouse chromosome 5, which mapped to four vole chromosomes (Figure 1). Conversely, vole chromosomes 2, 5 and 6 mapped to three mouse chromosomes each. To estimate the number of chromosomal rearrangements that would be necessary to generate the differences we observed between the genomes at this level of comparison we applied the GRIMM algorithm [17] to our comparative mapping data set. This method identified the five inversions described above, as well as 16 additional inter-chromosomal rearrangements, as the minimum number of rearrangements that would be needed to transform the marker order and chromosomal linkage in the prairie vole to that observed in the mouse genome. Previously it had been reported that there were fewer chromosomal rearrangements between the rat and deer mouse genomes compared to the mouse and deer mouse genomes [18]. As the prairie vole and deer mouse are sister species with respect to mouse and rat, we also used the GRIMM algorithm to estimate the minimum number of rearrangements detected in our data set between the prairie vole and rat. For the prairie vole-rat comparison in which the orthologous positions of the mapped prairie vole BAC clones in the rat genome were indirectly inferred via their orthologous location in the mouse genome using the UCSC Genome Browser 'convert' option [19], the GRIMM algorithm estimated a total of 37 rearrangements (3 inversions and 34 interchromosomal) between the prairie vole and rat. Thus, though the number of markers included in our comparative maps is relatively small, these observations suggest that the mouse genome could be a better reference for predicting the location of genes in the prairie vole genome versus the rat genome.

## Discussion

The prairie vole is an exceptional model of social behavior and is a member of a genus that has been associated with rapid evolution and atypical genomic features. The experimentally validated BAC library reported here will be a valuable resource for dissecting the genetic basis of social behavior and more generally, evaluating patterns and rates of evolution within *Microtus*. In particular, we have shown that the BAC library can be used for targeted comparative mapping of genes and regions of interest. Clones isolated from the library can be used for detailed cytogenetic mapping within and between *Microtus *voles, as well as templates for targeted genomic sequencing. In addition, the BAC-end sequencing described here is already being used to support the genome-wide identification of polymorphic genetic markers, such as SNPs and microsatellites, and global end-sequencing of the library would support the planned future high-quality assembly of the prairie vole genome http://www.genome.gov/10002154. Finally, the BAC library is also a resource for developing prairie vole BAC transgenic mice, or in the future transgenic voles [20], both of which will serve as powerful tools to experimentally validate the role of specific genes or alleles have in modifying social behavior.

Previous studies indicated that *Microtus *genomes are evolving rapidly [5-7]. Due to the relatively low resolution of our prairie vole-mouse comparative map, it is not possible to rigorously quantify the rate of evolutionary chromosomal rearrangements that have occurred in the prairie vole versus mouse lineages. For example, even though a minimum of 21 chromosomal rearrangements must have occurred to explain the differences in linkage between the mapped prairie vole BAC clones and their orthologous positions in the mouse genome, the most parsimonious evolutionary history for only four of those rearrangements could be reconstructed (using the human genome as an outgroup), and of those, an equal number are predicted to have occurred in the prairie vole and mouse lineages. Nevertheless, it is worth noting that as has been observed in comparisons between other mammalian genomes [21], conserved synteny was detected between the prairie vole and mouse genomes, although the low resolution of our current comparative map would not be able to detect changes in gene order within the regions of conserved synteny, nor the precise size of those regions. Thus, the gene linkage in the mouse, or other assembled rodent genomes, can be used as a reasonable estimate for inferring gene linkage in the prairie voles. Future studies comparing the genomes of the prairie vole and other rodents, including mouse, rat, and deer mouse, that utilize the BAC library reported here (or other methods) will facilitate the comprehensive characterization of the unique genomic features that have been of long-standing interest in the *Microtus *genus.

## Conclusions

The prairie vole has emerged as a powerful model organism to study the genetic and neurobiological underpinnings of social behaviors. As genetic studies in prairie voles and related vole species within the genus *Microtus *have been limited to a handful of candidate genes, the BAC library and the prairie vole-mouse comparative cytogenetic map that we describe here are an essential first step towards the development of a comprehensive suite of genomic resources for this species. These resources will be of enormous value for identifying new genes involved in social behaviors and in developing molecular and genetic tools to study the relationships between the genome, the brain and social behaviors. In addition, these resources provide a platform to further explore the unique aspects of genome evolution within the genus *Microtus *relative to other rodent lineages.

## Methods

### BAC library construction

Euthanasia and collection of kidney tissue from an adult male prairie vole were performed as per guidelines that were reviewed and approved by the Emory Institutional Animal Care and Use Committee and were conducted in accordance with the *Guide for Care and Use of Laboratory Animals *published by the National Research Council. A vole BAC library (CHORI-232) was constructed from frozen kidney tissue from a male following previously described methods [22]. Briefly, the frozen kidney tissue was ground to a fine powder, resuspended in chromatin isolation buffer then embedded in 0.5% InCert agarose. Proteins were removed by a detergent/proteinase K treatment and the resulting high molecular weight DNA was partially digested using a combination of EcoRI restriction enzyme and EcoRI methylase enzyme. The DNA was size fractionated by pulsed-field gel electrophoresis and DNA fragments from the appropriate size fraction were cloned into the pTARBAC2.1 vector between the two EcoRI sites. The ligation products were transformed into DH10B (T1-resistant) electro-competent cells (Invitrogen). The library was arrayed into 528 (384-well) microtiter dishes and gridded onto 11 22 × 22 cm nylon high-density filters for screening by probe hybridization (see http://bacpac.chori.org/library.php?id=481.

### Targeted physical mapping

The vole BAC library was screened with overgo-hybridization probes designed for screening rodent genomic libraries [23]http://uprobe.genetics.emory.edu/, or from published vole sequences, within or near 19 genes of interest. A single pool of overgo-probes consisting of two to four probes/locus were hybridized to the BAC library and probe-content and restriction-enzyme fingerprint maps of each targeted region were constructed using previously described methods [24]. The combined probe-content and restriction-enzyme mapping data was used to estimate the depth of coverage of the library by calculating the average number of BACs that hybridized to each probe. Note that instances of false-positive and non-specific hybridization were excluded from the estimation of the library depth by eliminating clones that fell in contigs other than those that were anchored to the orthologous position of the targeted intervals by BAC-end sequences (see below).

### BAC-end sequencing and comparative mapping

BAC-end sequences (BESs) were generated by the British Columbia Cancer Agency Genome Sciences Centre, Vancouver, Canada using BAC DNAs extracted by a modified alkaline lysis preparation in 384 well format and sequenced with the BigDye Terminator 3.1 cycle sequencing kit (Applied Biosystems) on ABI 3730 × l sequencers and the following primers: T7 (5'-taatacgactcactataggg-3'), and SP6 (5'-atttaggtgacactatag-3'). Quality-trimmed and repeat masked BESs were then mapped to the mouse genome assembly (mm8) using MEGABLAST (-t 16, -N 2, -W 11, -e 1e-10) [25]. Individual vole BESs were initially classified as either mapping to 0, 1 or >1 location(s) in the mouse genome. In cases where both mate-pair reads from a single BAC could each be mapped to a discrete location in the mouse genome, the orientation and distance between the mate-pair alignments was used to classify clones as 'concordant' (orientation: + and - strand; distance: 90-200 kb) or 'discordant' (orientation: + and + strand, or - and - strand; distance: < 90 kb or > 200 kb). To define the most-likely position of BESs that mapped to more than one location in the mouse genome, when applicable, the location of a uniquely mapped mate-pair was used to search the alternative genomic locations for one that would meet the criteria set for 'concordant' clones. All BESs have been deposited in GenBank (GenBank: FI596473-FI599626, FI846759-FI847487).

### Cell culture

Male prairie vole fibroblast cell cultures were established from a tissue homogenate produced by manual and enzymatic digestion using a protocol adapted from [26]. Kidney tissue was washed in 5 ml complete media [MEM (Invitrogen/Gibco, Carlsbad, California)] containing 10% heat-inactivated FBS (Invitrogen/Gibco, Carlsbad, California), 100 units/ml penicillin, and 100 mg/ml streptomycin (Invitrogen/Gibco, Carlsbad, California), manually minced, and resuspended in 0.5 ml PBS. Cells were incubated with collagenase B (final concentration 0.25% in PBS) for 15-30 minutes at 37°C. To ensure complete homogenization, the tissue suspension was suctioned through a Pasteur pipette. Cells were supplemented with 10 ml of complete media and incubated at 37°C.

### Metaphase chromosome preparations

When the cultures reached 80% confluency, cells were incubated with KaryoMAX Colcemid (100 ng/ml media) (Invitrogen/Gibco, Carlsbad, California) at 37°C for 5-6 hours. The cells were trypsinized from the surface of the flask using TrypLE Express (Invitrogen/Gibco, Carlsbad, California) for 10 min at 37°C. Cells were rinsed with 1.5 ml of media, centrifuged, and the pellet was suspended in 5 ml of 75 mM KCl hypotonic solution and incubated for 20 min at 37°C. The cells were then treated with 1 ml of methanol:glacial acetic acid (3:1) fixative, centrifuged, and resuspended in 10 ml fixative. This final step was repeated two times before metaphase slide preparation.

### Fluorescence in situ hybridization (FISH) analysis

A genome-wide set of prairie vole BACs were selected from clones anchored to the mouse genome via the BESs. Priority was first given to clones in which both BESs were anchored to the mouse genome and the remaining gaps in genome coverage were filled in with clones for which a single BES was anchored to the mouse genome. Prairie vole BAC DNA was isolated from overnight cultures with the appropriate antibiotic using an alkaline lysis procedure or an automated extraction system (Autogen, Inc., Holliston, MA). Fluorescently-labeled nucleotides [Spectrum Orange-dUTP, Spectrum Green-dUTP (Abbott Molecular Inc., Des Plaines, IL), or Diethylaminocoumarin-5-dUTP (PerkinElmer Life Sciences, Inc., Boston, MA)] were incorporated into the BAC DNA using a standard nick-translation (Abbott Molecular #07J00-001, Abbott Park, IL) or random priming reaction (Bioprime DNA labeling system, Invitrogen, #18094-011, Carlesbad, CA). Slides were washed in 2× SSC at 37°C for 30 minutes, and dehydrated sequentially in 70%, 80%, and 95% ice-cold ethanol. Chromosomes were denatured in 70% formamide/2× SSC at 75°C for 2 minutes, and then dehydrated as before. Prior to hybridization, probes were denatured at 75°C for 7 min and reannealed at 45°C for 1-10 minutes. Chromosome spreads were hybridized to probes overnight in a humid chamber at 37°C. Slides were washed in 0.4× SSC/0.3% NP-40 at 75°C for 2 minutes followed by washing in 0.2× SSC/0.1% NP-40 at room temperature for 30 seconds. Slides were mounted in VectaShield antifade solution with DAPI (Vector Laboratories, Burlingame, CA) and analyzed using digital-imaging with a CCD camera and software (SmartCapture 2, Digital Scientific, Cambridge, UK).

## Authors' contributions

LAM, LJY and JWT conceived and managed the project. JKD performed the targeted physical mapping and preparation of clones for BES. BH, MK and JKD constructed the BAC library. JJL and MKR performed the cytogenetic mapping. LAM, JWT and MKR drafted the manuscript and all authors approved the final version.

## Supplementary Material

Additional file 1 **Supplementary Table S1**. Genes and orthologous positions in the mouse genome targeted for physical mapping in vole. Click here for file

Additional file 2 **Supplementary Table S2**. Vole BAC clones mapped to mouse genome (mm8) by a single BES. Click here for file

Additional file 3 **Supplementary Table S3**. Concordant vole BAC-end mate pairs compared to mouse genome (mm8). Click here for file

Additional file 4 **Supplementary Table S4**. Discordant vole BAC-end mate pairs compared to the mouse genome (mm8). Click here for file

Additional file 5 **Supplementary Table S5**. Orthologous position of prairie vole BAC clones in the mouse genome (mm8) and chromosomal position in the prairie vole. Click here for file
